# Simpler and safer anastomosis by pancreaticogastrostomy using a linear stapler after pancreaticoduodenectomy

**DOI:** 10.1097/MD.0000000000040456

**Published:** 2024-11-08

**Authors:** Hirotaka Okamoto, Atsushi Yamamoto, Kenji Kawashima, Toshio Fukasawa

**Affiliations:** a Department of Surgery, Tsuru Municipal Hospital, Tsuru City, Yamanashi, Japan.

**Keywords:** pancreaticoduodenectomy, postoperative pancreatic fistula (POPF), stapled pancreaticogastrostomy

## Abstract

Postoperative pancreatic fistula (POPF) remains a major and serious problem after pancreaticoduodenectomy (PD). In its presence, pancreatic juice may leak from the main duct or branches of the pancreatic stump. To prevent this, we have applied a newly modified anastomosis of pancreaticogastrostomy (PG) using a linear stapler (stapled PG). Clinical records of 30 consecutive patients who underwent PD were reviewed between 2013 and 2023 at our community hospital. Regarding procedures, 12 stapled PGs and eighteen pancreaticojejunostomies (PJs) were performed after PD, from 2018 to 2023 and from 2013 to 2017, respectively. The pancreas was transected for long compression by a linear stapler, involving: pre-compression for 5 min, stapling for 5 min, and dissection for 5 min. After removal of the staples at the main duct opening of the pancreatic stump, PG anastomosis was performed. The outer layer was anastomosed by a straight single row pancreas-transfixing suture with the posterior gastric wall, and inner layer duct-to-mucosa anastomosis was also performed in a radial axis manner. Anastomosis of PJ was conducted without using a linear stapler. POPF was defined as a clinical manifestation of POPF (grade B/C) based on the ISGPF (International Study Group of Pancreatic Fistula) criteria. None of the 12 patients who had undergone stapled PG developed clinically relevant POPF, whereas 5 (27%) patients who had received PJ developed POPF. Three patients showed POPF grade B and 2 patients exhibited POPF grade C. Stapled PG after PD may reduce clinically relevant POPF. Because our sample size was small, the further accumulation of cases is required to validate this method.

## 1. Introduction

Although numerous studies have been reported on methods to prevent pancreatic fistula after pancreaticoduodenectomy (PD), the incidence of postoperative pancreatic fistula (POPF) remains high, at approximately 5% to 40%.^[1–3]^ The risk of POPF is increasing, especially in the presence of a soft pancreas, which markedly contributes to pancreatic juice leakage from the cut surface of the pancreas. The juice leaks from not only the main duct but also the duct branches, which do not drain into the main pancreatic duct.^[4–6]^

Various methods have been employed to prevent fistula development, including Blumgart anastomosis, duct-to-mucosa anastomosis with or without stenting, pancreaticogastrostomy (PG), or prophylactic somatostatin analogue administration.^[7–10]^ The effect of a linear stapler to prevent POPF was also reported in distal pancreatectomy (DP) cases.^[11]^ The technique was based on the theory that mechanical compression stapling could seal the ductules.

We have applied the tri-row linear stapler when transecting the pancreas during PG following PD, to prevent juice leakage from the pancreatic stump, especially from duct branches. Herein, we demonstrate our surgical technique of PG after stapled PD and report the outcomes.

## 2. Materials and methods

Thirty consecutive patients who underwent PD with pancreaticojejunostomy (PJ) or PG at the Department of Surgery, Tsuru Municipal Hospital, Japan, from 2014 to December 2023, were reviewed. Data were collected retrospectively from the hospital database. All 30 patients gave informed consent for surgical treatment. This study was conducted in accordance with the ethical principles outlined in the Declaration of Helsinki and was approved by the Institutional Review Board (approval no. 202404). Patients’ demographics are shown in Table 1, including age, sex, and BMI (body mass index). Primary diseases were as follows: distal bile duct cancer, papilla of Vater ampullary cancer, duodenal cancer, intraductal papillary mucinous neoplasia (IPMN), and neuroendocrine cancer (NEC). Pancreatic cancer cases in which patients had undergone PD were excluded because of their hard pancreatic texture. Patients’ physical status was expressed according to ASA (American Society of Anesthesiologists) physical status classification system.^[12]^

**Table 1:** Patient characteristics.

		Stapled PG	PJ	*P* value
N		12	18	
Age (yr ± SD)		72.4 ± 13 (54–88)	70.3 ± 11 (43–85)	.50
Sex (M/F)		5/7	2/1	.35
BMI (kg/m^2^)		20.8 (17.6–22.4)	22.8 (16.8–26.2)	.11
ASA	1	10	14	.68
	2	2	2	
	3	0	2	
	4	0	0	
	5	0	0	
Primary disease				
Cancer of ampulla		6	8	.45
Cancer of distal bile duct		2	4	
Cancer of duodenum		0	1	
IPMN		4	4	
NEC		0	1	
Operative procedure				
PPPD		2	10	.057
SSPPD		10	8	
Status of pancreas				
Thickness (mm ± SD)		13.2 ± 2.7	14.1 ± 2.1	.41
MPD diameter (mm ± SD)		4.3 ± 1.4	4.4 ± 1.8	.38

ASA = american society of anesthesiologists, IPMN = intraductal papillary mucinous neoplasia, MPD = main pancreatic duct, NEC = neuroendocrine carcinoma, PPPD = pylorus-preserving pancreaticoduodenectomy, SSPPD = subtotal stomach-preserving pancreaticoduodenectomy.

POPF was defined and classified according to the International Study Group on Pancreatic Fistula (ISGPF) definition, as follows: persistent drainage after 3 weeks, signs of infection, readmission within a month, or fluid collection with an elevated drainage level >3 times the normal serum level.^[13,14]^

The main pancreatic duct diameter was measured by preoperative computed tomography (CT) and/or ultrasound examination.

## 3. Surgical technique

**Figure 1.**
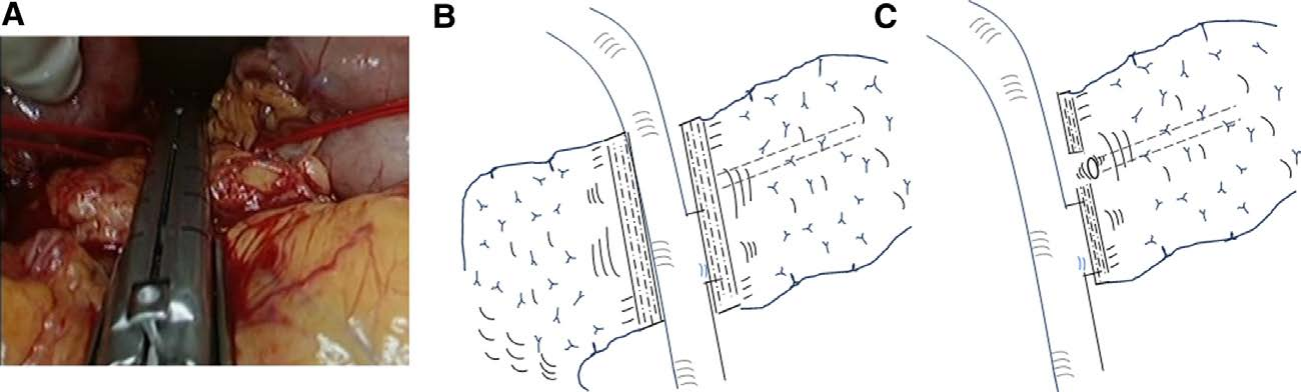
The pancreas was kept compressed and transected above the portal vein at the pancreatic neck by a linear stapler (A). Schematic view of the transected pancreas (B). Several staplers at the opening site of the main pancreatic duct at the stump were removed for pancreaticogastrostomy (C).

We transected the pancreas using a COVIDIEN tri-stapler (Tri-Stapler, Black 60 mm, COVIDIEN, MA, USA). The neck of the pancreas was dissected from surrounding tissue and tunneled from the superior mesenteric and portal veins. The pancreas was transected by the stapler above the portal vein at the neck during PD. Firstly, the pancreas was gently compressed directly by intestinal clamp forceps (B. Braun Aesculap Co., Ltd., Melsungen, Germany) for 5 min. After this first compression, the pancreas was compressed by Endo-GIA for another 5 min. Subsequently, the pancreas continued to be compressed and was dissected for a further 5 min. Then, the transection was completed (Fig. 1A and B). Prior to reconstruction of PG after PD, several staplers at the opening site of the pancreatic duct were removed by scissors (Fig. 1C). In some cases, in which the position of the main duct was unclear, intraoperative ultrasonography was performed to confirm its location.

A remnant pancreas of about 2 cm in length was dissected to mobilize from the surrounding tissues in preparation for PG anastomosis. A serosal muscular incision, about the same size as the remnant pancreatic stump, was made in the posterior wall of the stomach, and a mucosal incision of about 2 to 3 mm was also made in order to insert a 4 to 6 Fr polyethylene pancreatic stent tube (Sumitomo Bakelite Co., Tokyo, Japan). After setting an appropriate tension-free position of the remnant pancreas with the stomach, PG was started.

**Figure 2.**
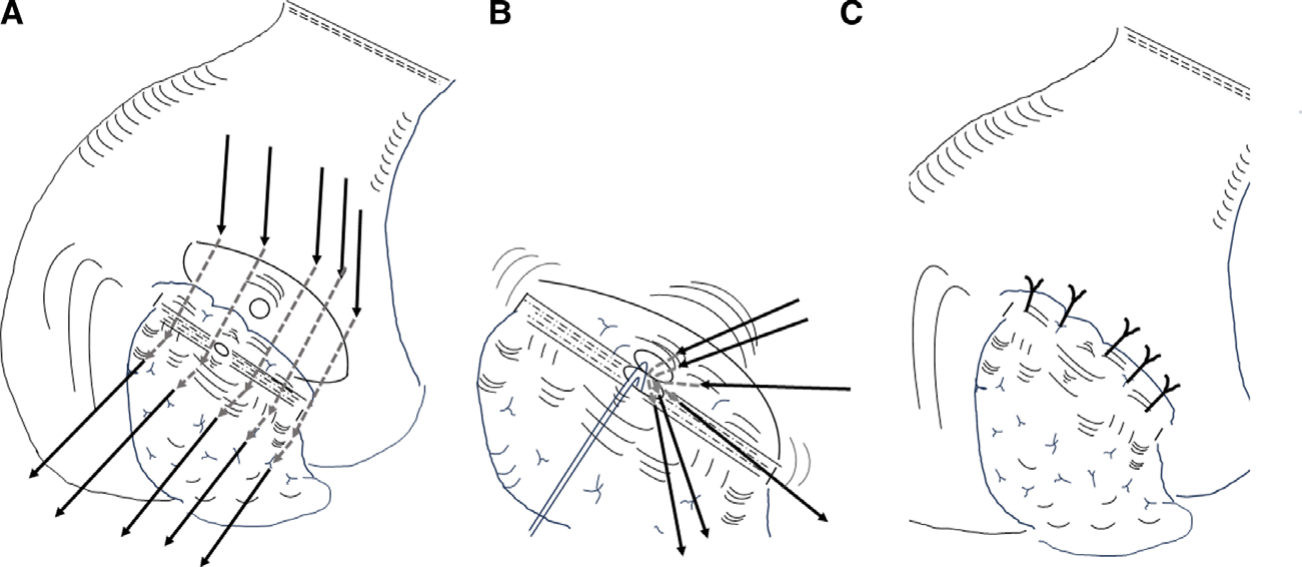
(A) A serosal muscular incision with the same size as the remnant pancreatic stump was placed in the posterior wall of the stomach, and mucosal incision of about 2 to 3 mm was also made in order to insert a 4 to 6 Fr polyethylene pancreatic stent tube. (B) Pancreatic duct-to gastric mucosa anastomosis was also performed. Insertion of the pancreatic tube into the main duct and introduction of the opposite end of the tube into the stomach cavity via the mucosal incision hole. In total, 8 duct-to-mucosa sutures were applied in a radial axis manner. (C) Five to 6 straight transfixing sutures between the posterior gastric wall and remnant pancreatic parenchyma were placed to embed the stapled pancreatic stump with gastric wall.

A 3-0 monofilament polyproplene thread of 26 mm (half circle, taper point needle (Prolene, Ethicon Inc., NJ)) was used for straight transfixing suture between the gastric posterior wall and remnant pancreatic parenchyma. The needle was handled, starting from the distal posterior gastric wall to proximal posterior wall, at the serosal-muscle depth. Thereafter, the needle was gently passed from the ventral to dorsal surfaces of the remnant pancreas at a point approximately 1.5 cm caudal from the stump edge, carefully avoiding needle penetration into the main pancreatic duct. About 5 to 6 sutures were placed to embed the stapled pancreatic stump with gastric wall (Fig. 2A).

Pancreatic duct-to gastric mucosa anastomosis was also performed using a 5-0 absorbable monofilament polydioxanone thread of 11 mm [half circle, taper point needle (PDS-II, Ethicon Inc.)]. The needle was handled, starting from the proximal gastric mucosa to ventral pancreatic duct, with 5 sutures. The insertion of the pancreatic tube into the main pancreatic duct and introduction of the opposite end of the tube into the stomach cavity via the mucosal incision hole were performed. Thereafter, the needle was carefully handled from the distal gastric mucosa to dorsal pancreatic duct with 3 sutures to complete duct-mucosal anastomosis. Duct-to-mucosa sutures were completed to ligate in a radial axis manner (Fig. 2B).

Finally, 5 to 6 already prepared sutures coming from the gastric distal posterior wall with sutures from the remnant pancreatic dorsal parenchyma were gently ligated to complete PG (Fig. 2C). If reinforcing sutures were necessary, additional sutures were applied to both the upper and caudal edges of the pancreatic parenchyma with gastric wall.

Hepaticojejunostomy (HJ) in an end-to-side fashion and subsequently gastrojejunostomy (GJ) in an end-to-side fashion approximately 40 cm distal to the site of HJ anastomosis were performed in an antecolic fashion. An enteral feeding tube was introduced via the jejunal stump to manage the delayed gastric emptying. The pancreatic duct tube was cut to remain in the gastric cavity for use of the lost stent tube. To manage the pancreatic fistula, a drainage tube was inserted to around the PG site in the posterior space of the stomach, which was removed around day 3 after surgery in most cases.

With the conventional PJ method, the pancreas was transected directly with a surgical scalpel without the stapler after tunneling the pancreatic neck above the portal vein. Pancreatic duct-to jejunal mucosa anastomosis was performed by 8 applying sutures resembling a radial axis. Anastomosis of the stump of the pancreatic parenchyma to jejunal serosal-muscle was also carried out. PJ was performed in an end-to-side fashion approximately 10 cm distal to the jejunal stump. Subsequently, HJ was also performed in an end-to-side fashion 6 to 8 cm distal to the PJ anastomosis. Finally, GJ in an end-to-side fashion was conducted (antecolic fashion) approximately 40 cm distal to the HJ anastomosis.

The right gastroepiploic vessels were divided at their origin while preserving the gastroepiploic arcade, and the right gastric artery was also divided at the origin. The duodenum was transected about 2 to 3 cm distal to the pylorus in the case of pylorus-preserving PD (PPPD), while the antrum of the stomach was transected about 3 to 4 cm proximal to the pylorus in the case of subtotal stomach-preserving PD (SSPPD) in both stapled PG and PJ cases.

Statistical analysis was performed using Chi-square statistics and the Mann–Whitney *U* test to evaluate patient characteristics and postoperative outcomes in the 2 groups by SPSS version 22.0. Significance was defined as *P* < .05.

## 4. Results

Patient characteristics are shown in Table 1. Their age, sex, ASA physical status classification, primary disease, and operative methods were extracted. The status of the pancreas, such as its thickness and the main pancreatic duct diameter at the neck above the portal vein, was also recorded. These characteristics were compared between the stapled PG and PJ groups.

There were no significant differences in terms of age, sex, BMI, or ASA score. Numbers of primary diseases resulting in the surgery in stapled PG and PJ groups were as follows: distal bile duct cancer: 6 and 8; ampullary cancer: 2 and 4; duodenal cancer: 0 and 1; intraductal papillary mucinous neoplasia (IPMN): 4 and 4; neuroendocrine carcinoma (NEC): 0 and 1, respectively.

The technique for PG after pancreatic head resection using the tri-stapler was performed in 12 consecutive cases during the period between 2018 and 2023. This stapled PG group was assessed by comparing with our conventional PJ group between 2013 and 2017. Numbers of operative procedures were 2 in PPPD and 10 in SSPPD in the stapled PG group, and 10 in PPPD and 8 in SSPPD in the PJ group, respectively. Parameters associated with POPF such as pancreatic thickness and the main pancreatic duct are shown. There were no hard pancreatic texture cases. Also, there were no significant differences between stapled PG and PJ groups.

Amylase levels in drainage fluid obtained from a drain around anastomosis sites were assessed on postoperative day (POD) 3 and 7. Table 2 shows these levels on POD 3 in stapled PG and PJ groups. Amylase values in the stapled PG group were lower than those in the PJ group, showing a significant difference (*P* < .05). All cases showed mostly a normal soft pancreatic texture, subsequently confirmed by pathological examination.

**Table 2:** Perioperative outcomes.

	Stapled PG	PJ	*P* value
D-amylase on POD3 (U/L)*	536 ± 725	4507 ± 3191	<.05
POPF grade			
B	0	3	<.001
C	0	2	
Morbidity (Hemmorhage, Pneumonia, Abscess)	0	5	<.001
Mortality	0	0	

* Amount of amylase in drainage fluid drains positioned near the anastomosis site on postoperative day 3 (POD3), POPF: Postoperative pancreatic fistula.

POPF was not observed in the stapled PG group, but was present in 5 patients in the PJ group (3 grade B patients, and 2 grade C patients) (Table 2), and this difference was significant (*P* < .001). All 3 patients with POPF grade B in the PJ group required continual drainage for more than 3 weeks with infection signs. Regarding the 2 patients with POPF grade C in the PJ group, 1 required continual drainage for more than 3 weeks since drainage fluid was contaminated with signs of infection and abdominal fluid collection on CT; the other patient required interventional radiological therapy, such as for angio-embolism, due to unexpected bleeding from the drainage tube on POD 10. The patient recovered uneventfully following the interventional therapy.

## 5. Discussion

We applied stapled PG after PD, which is simple, safe, and promotes a reduced rate of POPF. The method of stapled PG has shown the clinical benefits of POPF prevention and a shorter drainage period. The cause of POPF is considered to involve several factors, 1 of which is pancreatic juice leakage from the cut surface, particularly from the branch duct as well as main duct. Also, juice leakage from small holes created by the curved needle was pointed out. Therefore, we applied the stapler for PG after PD in order to prevent these causes of juice leakage. To reduce leakage from the pancreatic stump, we considered the possibility of using a stapler. There is accumulating evidence to support reduction of POPF with the use of a linear stapler in DP cases. The technique of prolonged prefiring compression of the pancreas using a stapler is effective, because the mechanical stapling method can seal the ductules without causing injury.^[11]^

There are no reports on linear stapler use for PD. We used a stapled remnant pancreas for PG with a tri-row stapler. This enabled any tissue damage to be minimized. The beneficial effects are considered to be due to its graduated reduced compression with clamping and the potential for greater perfusion into the staple line toward applied tissue.^[15]^ Maintaining tissue blood supply by these compression and perfusion techniques might reduce damage to the pancreas on transection with complete clamping of the duct branches without tissue necrosis. Eventually, this will lead to reduced juice leakage. Furthermore, we used a prolonged compression clamping technique to reduce tissue damage. Pre-compression by clamping forceps, compression, and firing with Endo-GIA for 5 min, respectively, were performed. This procedure might help minimize injury and damage of fragile pancreatic tissue.

We have switched to PG from PJ after PD. This has mainly been because of the advantages of: the absence of enterokinase, relatively thick gastric wall of the stomach, and simplicity of the straight transfixing suture. One possible cause of POPF was considered to be enterokinase in jejunal fluid activating the leaked pancreatic juice, resulting in worsening PF severity.^[16,17]^ PG could prevent POPF from worsening because of the absence of enterokinase and low pH in the stomach, even if PF was relevant. Another reason to select PG is the simplicity of its anastomosis.^[18]^ We have applied straight transfixing suture between the posterior gastric wall and pancreas parenchyma with some modifications.^[19,20]^ In our study, this technique could be uneventfully performed by an inexperienced operator in our department. One of the disadvantages pointed out was a slightly impaired surgical view, but we cannot avoid such a difficult situation. Simple straight row transfixing sutures could reduce damage of fragile pancreatic parenchyma as much as possible. Our result of a low rate of POPF is mainly not only due to reduced juice leakage from the stapled pancreas but also minimal trauma facilitated by simple straight suturing.

Duct-to-mucosa sutures with a lost internal stent were performed in a radial axis manner in both PG and PJ cases. In a previous report, duct-to-mucosa anastomosis was recommended to avoid obstruction of the pancreatic duct and/or subsequent atrophy.^[21]^ With regard to PG, a problem with anastomosis patency of the pancreatic duct arose on long-term follow-up. Stricture of the anastomosis led to exocrine or endocrine problems, resulting in malabsorption or diabetes. In our cases, despite the duct-to-mucosal anastomosis, 3 patients with stapled PG developed duct dilatation. However, clinically relevant exocrine dysfunction or diabetes was not noted in the follow-up period. Our experience was not long, and so further follow-up is necessary.

In conclusion, stapled PG with straight row transfixing and duct-to-mucosa anastomosis after PD may be a simple, safe, and reliable method, leading to a reduction of clinically relevant POPF. Because this study was small and preliminary, we should evaluate the present technique involving a larger prospective series.

## Author contributions

**Conceptualization:** Hirotaka Okamoto.

**Data curation:** Hirotaka Okamoto.

**Formal analysis:** Hirotaka Okamoto.

**Investigation:** Hirotaka Okamoto, Atsushi Yamamoto.

**Methodology:** Hirotaka Okamoto, Atsushi Yamamoto.

**Supervision:** Hirotaka Okamoto, Atsushi Yamamoto.

**Validation:** Hirotaka Okamoto, Atsushi Yamamoto, Kenji Kawashima, Toshio Fukasawa.

**Visualization:** Hirotaka Okamoto.

**Writing – original draft:** Hirotaka Okamoto.

**Writing – review & editing:** Hirotaka Okamoto.
